# Silvopastoral systems benefit invertebrate biodiversity on tropical livestock farms in Caquetá, Colombia

**DOI:** 10.1111/afe.12594

**Published:** 2023-10-10

**Authors:** Lois Kinneen, María Paula Escobar, Luis Miguel Hernandez, Jill Thompson, Yardany Ramos‐Pastrana, Eric Córdoba‐Suarez, Miguel Romero‐Sanchez, Andrew Barnes, Marcela Quintero, Michael P. D. Garratt

**Affiliations:** ^1^ Sustainable Land Management, School of Agriculture, Policy & Development University of Reading Reading UK; ^2^ Bristol Veterinary School University of Bristol Bristol UK; ^3^ Alliance of Bioversity International and International Center for Tropical Agriculture (CIAT) Cali Colombia; ^4^ UK Centre for Ecology & Hydrology Midlothian UK; ^5^ Universidad de la Amazonia, Grupo de Investigación en Entomología Universidad de la Amazonia (GIEUA), Laboratorio de Entomología (LEUA) Florencia, Caquetá Colombia; ^6^ Department for Rural Economy Environment and Society Edinburgh UK

**Keywords:** agroecology, malaise traps, silvopasture, sustainable agriculture, sweep netting, tropical ecology

## Abstract

1. In the Colombian Amazon, there has been long‐term and sustained loss of primary forest threatening biodiversity and climate change mitigation. Silvopastoral practices that integrate trees into livestock production could help address both local economic and wider environmental challenges.
2. We aimed to assess the effects of silvopastoral practices on invertebrate communities on smallholder farms in Caquetá, Colombia. Using sweep nets and malaise trapping, invertebrate communities were compared between traditional pasture, silvopasture and forest edge habitats.
3. Invertebrate communities collected using sweep nets were contrasting among habitat types, communities were significantly different between traditional pasture and forest edge habitats and diversity and evenness were greatest in forest edges compared to traditional pastures. It appears that silvopasture areas, by supporting similar invertebrate assemblages to both traditional pasture and forest edges, may be acting as an intermediate habitat.
4. When individual invertebrate orders were compared, Lepidoptera and Coleoptera were found in greater abundance in the forest edge habitats, while Hemiptera were more abundant in traditional pasture. Hemipterans are often pests of forage plants in pasture systems and these differences in abundance may have implications for ecosystem services and disservices.
5. Silvopastoral approaches cannot replace the unique biodiversity supported by native forests but could deliver benefits for invertebrate conservation and ecosystem services if integrated into landscapes.

## Abstract (es)

**Resumen:**

1. En la Amazonía colombiana ha habido una pérdida sostenida y a largo plazo de bosque primario que amenaza la biodiversidad y la mitigación del cambio climático. Las prácticas silvopastoriles que integran los árboles en la producción ganadera podrían ayudar a abordar tanto los desafíos económicos locales como los ambientales.
2. Nuestro objetivo fue evaluar los efectos de las prácticas silvopastoriles en comunidades de invertebrados en pequeñas fincas en Caquetá, Colombia. Las comunidades de invertebrados se compararon entre las pasturas nativas, el sistema silvopastoril y los hábitats del borde del bosque mediante el uso redes entomológicas de barrido y trampas Malaise.
3. Las comunidades de invertebrados recolectadas usando redes entomológicas de barrido contrastaban entre los tipos de hábitat. Las comunidades eran significativamente diferentes entre las pasturas nativas y el borde de bosque. Pareciera que las áreas de silvopastoreo, al soportar conjuntos de invertebrados similares tanto a las pasturas nativas como a los bordes del bosque, pueden estar actuando como un hábitat intermedio.
4. Cuando se compararon los órdenes individuales de invertebrados, los lepidópteros y los coleópteros se encontraron en mayor abundancia en los hábitats del borde del bosque, mientras que los hemípteros eran más abundantes en las pasturas nativas. Los hemípteros son a menudo plagas de plantas forrajeras en sistemas de pastoreo y estas diferencias en abundancia pueden tener implicaciones y perjuicios para los servicios ecosistémicos.
5. Los sistemas silvopastoriles no pueden reemplazar la biodiversidad única apoyada por los bosques nativos, pero podrían ofrecer beneficios para la conservación de invertebrados y los servicios ecosistémicos si se integran en los paisajes.

## INTRODUCTION

Global livestock farming has steadily increased over the last decades, particularly in developing countries, and this has resulted in significant environmental degradation, including widespread deforestation (Armenteras et al., 2013; Ilea, 2009). Deforestation continues to increase in the Amazonian regions of Colombia where it can be twice that of the continental average (González‐González et al., 2021; Murad & Pearse, 2018). While this deforestation is driven by multiple socio‐political and economic factors (Armenteras et al., 2013; Krause, 2020), it results in significant negative outcomes including loss of biodiversity, desertification and increased greenhouse gas (GHG) emissions (Chakravarty et al., 2012). We urgently need land management solutions that mitigate these negative impacts and as a result, there are many public and private initiatives throughout the world, aimed at reducing deforestation or promoting afforestation, including in Colombia (Furumo & Lambin, 2020; González‐González et al., 2021; Suárez Delucchi et al., 2022).

One such solution is the establishment of silvopasture, which aims to integrate trees and livestock into a single production system (Jose et al., 2019; Sales‐Baptista & Ferraz‐de‐Oliveira, 2021). There are numerous potential benefits of adopting silvopasture in place of more traditional treeless pastures such as enhanced forage production and forage quality, improved livestock productivity as well as environmental benefits such as biodiversity protection and carbon sequestration (Jose & Dollinger, 2019). As a result, many national and international initiatives provide support for farmers to adopt silvopasture practices for local and wider societal benefits (Suárez Delucchi et al., 2022). While the benefits of silvopasture systems from a production and GHG mitigation perspective are well‐studied (Jose & Dollinger, 2019), effects on biodiversity, including invertebrates, are less understood (Pinheiro & Hunt, 2020).

Invertebrates are essential components of biodiversity in most ecosystems, playing key functional roles from decomposition and recycling to herbivory and predation (Prather et al., 2013). This is very apparent for human‐modified agroecosystems, where altered invertebrate communities, in response to land use change, can have negative effects on production such as through increased invertebrate pests and parasites (Derek Scasta, 2015; Karp et al., 2018). Alternatively, modified land management can increase ecosystem service benefits delivered by invertebrates through improved pollination of crops and pest control by natural enemies (Dainese et al., 2019; Martin et al., 2019). Due to these potential effects on ecosystem services, it is important to understand the outcomes on invertebrate communities of transitioning from a business as usual forage only pasture to a silvopastoral system (Duran‐Bautista et al., 2020). Particularly, for invertebrate groups that have important functional roles within an agroecosystem such as pests and parasites for livestock production.

Invertebrates can act as a useful indicator or proxy for wider biodiversity (Paoletti, 2012), or changes in their populations may help identify ecological characteristics of a habitat or allow the detection of the effects of habitat management (Gerlach et al., 2013). From a conservation perspective, it is necessary to consider how invertebrate communities found in silvopasture compare to those found in more natural areas such as forest traditionally associated with high biodiversity. Thus, providing an indication of the biodiversity value of a transition to a silvopastoral system.

This study aimed to explore the extent to which invertebrate communities differ between habitats found on smallholder livestock farms in a deforested area of the Amazon and investigate how invertebrate communities in silvopastoral systems compare to more natural forest habitats and the traditional pasture areas they are proposed to replace.

## MATERIALS AND METHODS

### Study area and sampling design

This study was carried out on five livestock farms in the department of Caquetá in the Colombian Amazon (Figure S1). This area experiences a tropical climate, with a mean annual temperature of 25°C and mean annual rainfall of 3600 mm, with most rainfall occurring between April and November and a dryer season from December to March (Duran‐Bautista et al., 2020). The landscape is classed as ‘Lomerío’ (hilly) and comprises flat to undulating land, where the predominant land use is pasture.

The farms selected for this study were involved in the ‘Sustainable Amazonian Landscapes’ (SAL) project (Quintero et al., 2015), which investigated the impacts of implementing agri‐environmental initiatives, including silvopasture, on farm productivity, farmer livelihoods and some aspects of biodiversity including soil macrofauna. In our study, we looked at aboveground invertebrates on farms that had planted areas of silvopasture in 2016 where trees were planted in rows within pasture plots primarily composed of African grasses such as Brachiaria sp. (Landholm et al., 2019). Tree species planted included *Cariniana pyriformis* Miers, *Minquartia guianensis* Aubl., *Piptocoma discolor* (Kunth) Pruski, *Calycophyllum spruceanum* (Benth.) K.Schum., *Cedrela odorata* L., *Gmelina arbore* Roxb. and *Cordia alliodora* (Ruiz & Pav.) Oken in different combinations depending on the farms and were planted in rows across the silvopasture plots to create smaller paddocks where cattle grazing could be rotated. Study farms had a mean area of 64.2 ha (min. 20 ha max. 150 ha). Silvopasture plots within each farm formed a single block ranging in size from 0.5 ha to 3.7 ha. Across the farms, the largest land use type was traditional pasture, which generally formed a continuous land use area across the farms and was often heavily degraded due to grazing pressure. All of the farms also had small areas of remnant or secondary forest (see Figure S1 for total area of each habitat on each farm). In return for project help to establish, monitor and support, the silvopasture the farmers agreed to conserve these areas of forest and allow natural regeneration of forest on other areas of their farms.

### Invertebrate sampling

Fieldwork was carried out in July 2018, and two sampling methods were employed to survey invertebrate communities at each farm; sweep netting and malaise traps. On each farm, three habitat types were sampled including traditional pasture, silvopasture and forest edge habitats. Sweep netting was carried out along three 50 m transects located in each of the three habitat types on each farm totalling 45 transects across the five farms. A total of 50 sweeps were performed along each transect. Two rounds of sweep net sampling were performed on each transect across the farms approximately 2 weeks apart; however, for 12 of the 45 transects, the second round of sampling was not possible due to heavy rain. Transects were spread, as much as possible, across the habitat type on each farm to collect a representative sample of the invertebrate communities from each habitat. Forest edge transects were within 25 m and parallel to the forest edge. Due to variations in the size and extent of each habitat type across the farm, the silvopasture transects were often relatively close together, clustered in the areas of silvopasture established on each farm. Similarly, some forest edge transects were associated with different edges of a single patch of suitable forest on the farm.

Malaise traps were also set up on each farm, with one malaise trap in each of the traditional pasture and silvopasture areas collocated with one of the sweep net transects. A third malaise trap was set up in a patch of forest, at least 20 m inside the forest from the forest–pasture boundary. The malaise traps were left in place for 7 days. All invertebrate specimens were collected by sweep nets, and malaise traps were collected and stored in 70% ethanol and later identified to order level by the Grupo de Investigación en Entomología at the Universidad de la Amazonia.

### Statistical analysis

All analyses were carried out using the software R version 4.2.2 (R_Core_Team, 2022).

### Order richness, diversity and evenness

For each sampling method, a matrix of abundance data at each sampling location (either sweep transect or malaise trap) was produced. Order richness, Shannon diversity index and Pielou's evenness were then calculated using the R Package *vegan* (Oksanen et al., 2022). For the sweep netting data, mixed models were used to test for differences among these metrics across the habitat types using the R package lme4 (Bates et al., 2015). Round was nested within farm as random effects. For, order richness, a Poisson error distribution was assigned, and for Shannon diversity index and Pielou's evenness, Gaussian models fit adequately. Model fit was checked visually by inspecting residuals versus fitted values and histograms of residuals (Zuur et al., 2009). To make comparisons between the three habitat types, the R package *emmeans* (Lenth, 2023) was used to gather Tukey‐adjusted p‐values. Differences were considered significant with a 95% confidence interval when p‐values were less than 0.05. For the malaise data, since a single trap was set in each of the habitat types across the five farms, and since response data were not normally distributed, nonparametric Kruskal–Wallis tests were used.

### Invertebrate communities across different habitat types

To visualise the differences between invertebrate communities across the different habitats on each farm, nonmetric multidimensional scaling (NMDS) ordination was used. Here, for the sweep netting data, we used average abundance across sampling rounds when building our matrix to account for uneven sampling intensity. We then used the *metaMDS* function in the *Vegan* package (Oksanen et al., 2022) using Bray–Curtis distances. Ordination plots were produced using the R package *ggplot2* (Wickham, 2016). Polygons were added to the plots to indicate invertebrate assemblages found in each of the three habitat types.

For the sweep net samples, data on Isoptera were removed as this order was found only once across all 45 transects. For the malaise data, Archaeognatha, Isoptera and Thysanoptera were excluded as these were only found in 1 of the 15 malaise traps. These so‐called singletons provide little information about the effects of different habitats on biodiversity (Warton et al., 2015).

To analyse the differences between the invertebrate communities found in different habitats, we used the function *manyglm* from the *mvabund* package (Wang et al., 2012). This approach fits multivariate generalised linear models to a community response variable (Wang et al., 2012). By using this regression framework, offsets and blocking factors can be built into the models to account for experimental design, and different error distributions can be assigned to ensure model fit. In our case, for both the sweep and malaise analyses, we included farm as a blocking factor. For the sweep netting data, we also included an offset for the number of sampling rounds to account for variation in sampling effort. Model fit was assessed by visually inspecting Dunn–Smyth residuals plotted against fitted values. A negative binomial error distribution was selected for both the sweep and malaise models. Univariate hypothesis testing was then applied to determine which orders of invertebrate showed significant differences in abundance between habitat types. To further understand the differences in orders for which significant effects of habitat type were detected, mixed effects models and post hoc comparisons were made using lme4 and emmeans respectively.

To compare invertebrates collected by the two sampling methods, we created a binary presence/absence matrix and included a column for sampling method. The matrix was structured to include a single row for each habitat type at each of the five farms, meaning an order was marked as present if it had been caught along any of the three transects over the two rounds for each of the three habitat types. Again, we used the function *manyglm* from the *mvabund* package (Wang et al., 2012), and model fit was assessed by visually inspecting Dunn–Smyths residuals plotted against fitted values. For this analysis, a binomial error distribution was selected to account for the binary presence/absence data.

## RESULTS

Over the course of the sampling period, 10,782 individual invertebrate specimens were collected through sweep netting, including insects from 14 different orders, as well as arachnids such as spiders and mites. The most abundant order found in the sweep net samples was Hemiptera (3833) followed by Diptera (3145) and Araneae (1471) (Figure S2). In the malaise traps, 2935 specimens from 15 orders were collected. In contrast to sweep netting, for the malaise trap collections, Diptera (1481) and Hymenoptera (656) were most abundant (Figure S3). NMDS comparisons confirmed significant contrast between the communities samples by each method (Figure S4).

### Diversity, richness and evenness between habitat types

**FIGURE 1:**
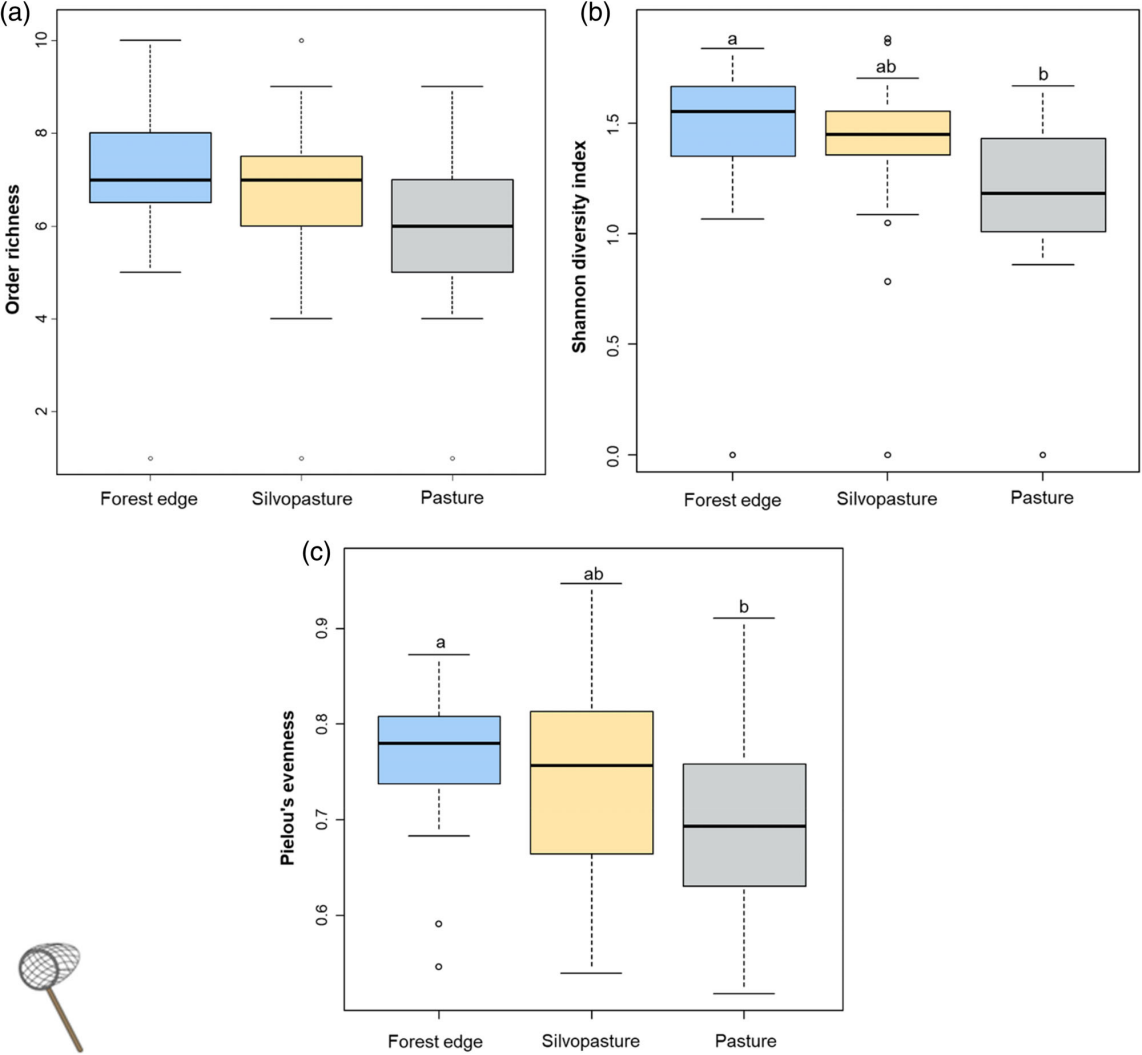
Boxplots depicting (a) order richness, (b) Shannon diversity index and (c) Pielou's evenness of invertebrate communities sampled using sweep netting according to habitat type. Different letters indicate significant difference (*p* ≤ 0.05) between habitats.

**FIGURE 2:**
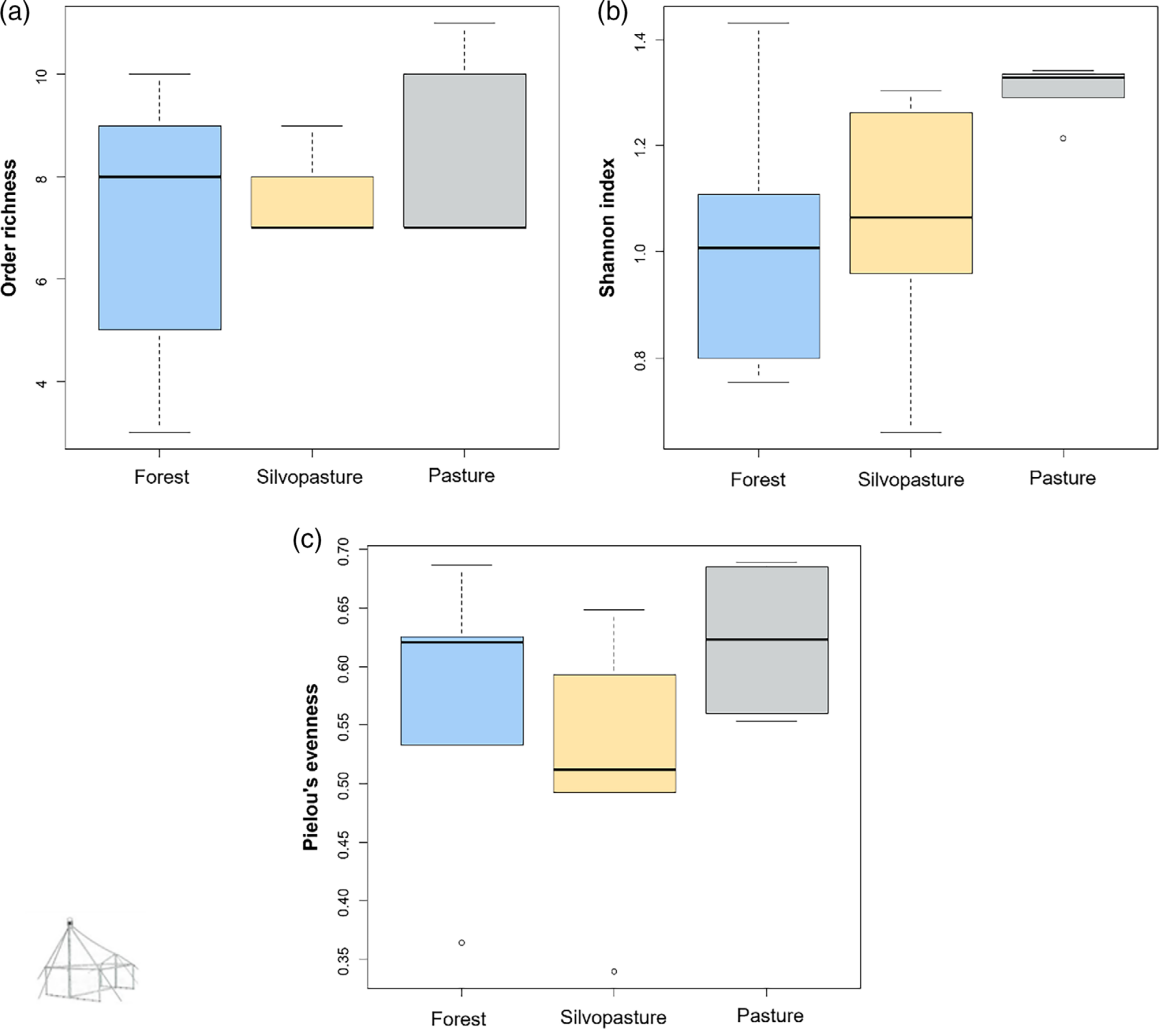
Boxplots depicting differences in a) Order richness, b) Shannon Diversity Index and c) Pielou's evenness of invertebrate communities sampled using malaise traps according to habitat type. No significant differences were found between habitat types for any of these metrics.

Communities of invertebrates sampled using sweep nets did not differ in terms of order richness (Figure 1a, Table S1) but significant differences were detected between forest and pasture communities in terms of diversity (pairwise post hoc comparisons, estimate = 0.26, *p* = 0.03, Table S1, Figure 1b) and evenness (pairwise post hoc comparisons, estimate = 0.07, *p* = 0.04, Table S1, Figure 1c). For the invertebrate communities collected using malaise traps, no significant differences in order richness (χ^2^ = 0.40, *p* = 0.82, Table S2, Figure 2a), Shannon diversity (χ^2^ = 4.34, *p* = 0.12, Table S2, Figure 2b) or Pielou's evenness (χ^2^ = 2.66, *p* = 0.26, Table S2, Figure 2c) were found.

### Comparing invertebrate assemblages among habitat types

**FIGURE 3:**
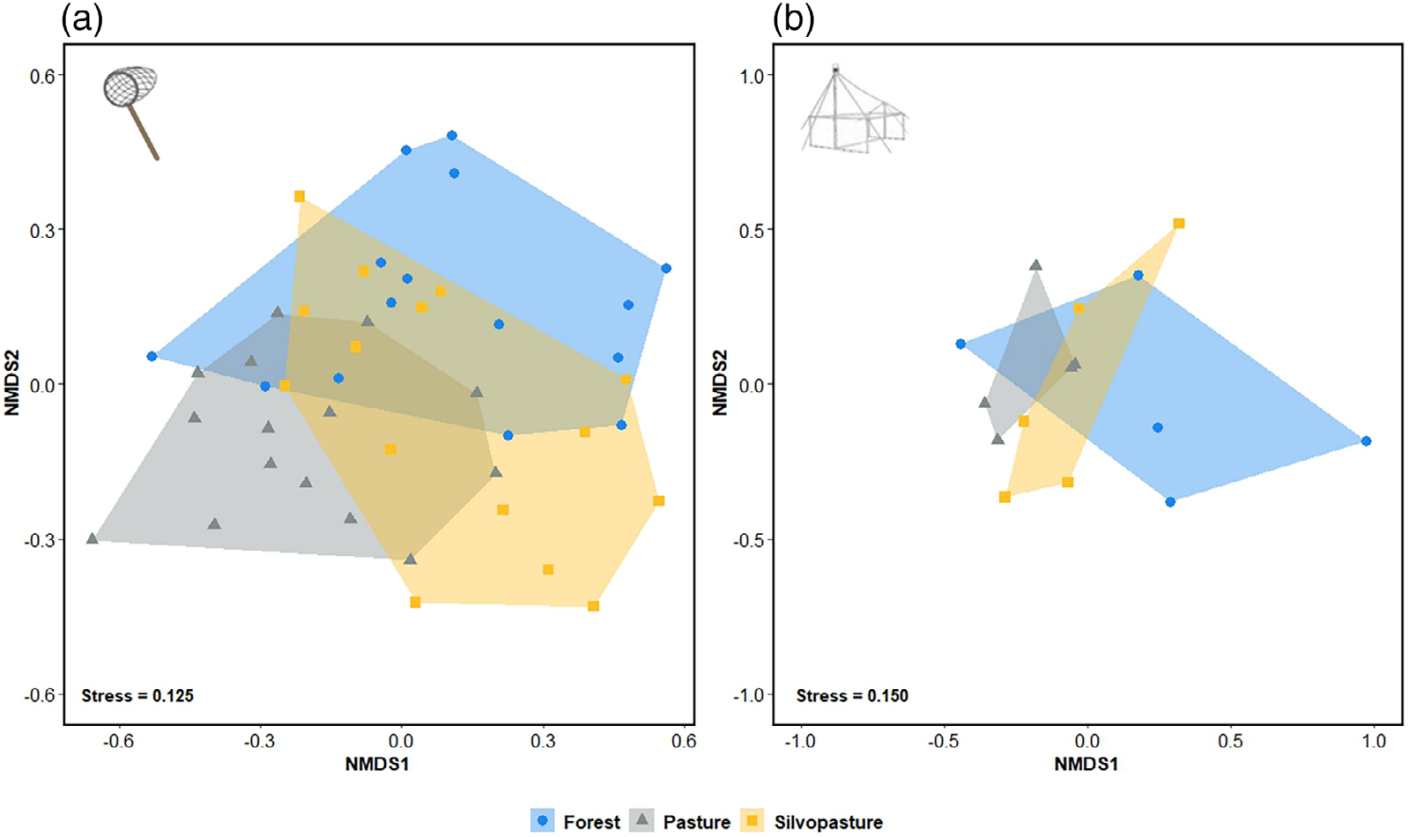
Non‐metric multidimensional scaling (NMDS) plots representing invertebrate communities collected using (a) sweep nets (on 45 transects) (k = 3, stress = 0.1245) or b) in 15 malaise traps in three different habitat types on five smallholder livestock farms in Caquetá, Colombia. For the sweep nets, points represent the invertebrate assemblages at each transect averaged across two sample rounds (where two rounds were available). For the malaise traps, each point represents the invertebrate communities sampled at an individual trap. Points closer together represent assemblages that are more similar than those further apart in ordination space.

Invertebrate communities sampled through sweep netting in the three habitats were significantly different (Deviance = 78.02, d.f. = 42, *p* = 0.001, Table S4). While there was considerable overlap in invertebrate communities among habitats as can be seen in the NMDS plots, the polygons were different (Figure 3a), and according to pairwise comparisons, communities were significantly different between forest edge habitats and traditional pasture (Observed statistic = 48.26, d.f. = 42, *p* = 0.013, Table S4). Differences between forest edge and silvopasture (Observed statistic = 28.90, d.f. = 42, *p* = 0.072) and silvopasture and traditional pasture were not significant (Observed statistic = 33.63, d.f. = 42, *p* = 0.071, Table S4). Habitat type did not have a significant impact on invertebrate communities collected through malaise trapping (Table S5).

### Order level differences among habitats

**FIGURE 4:**
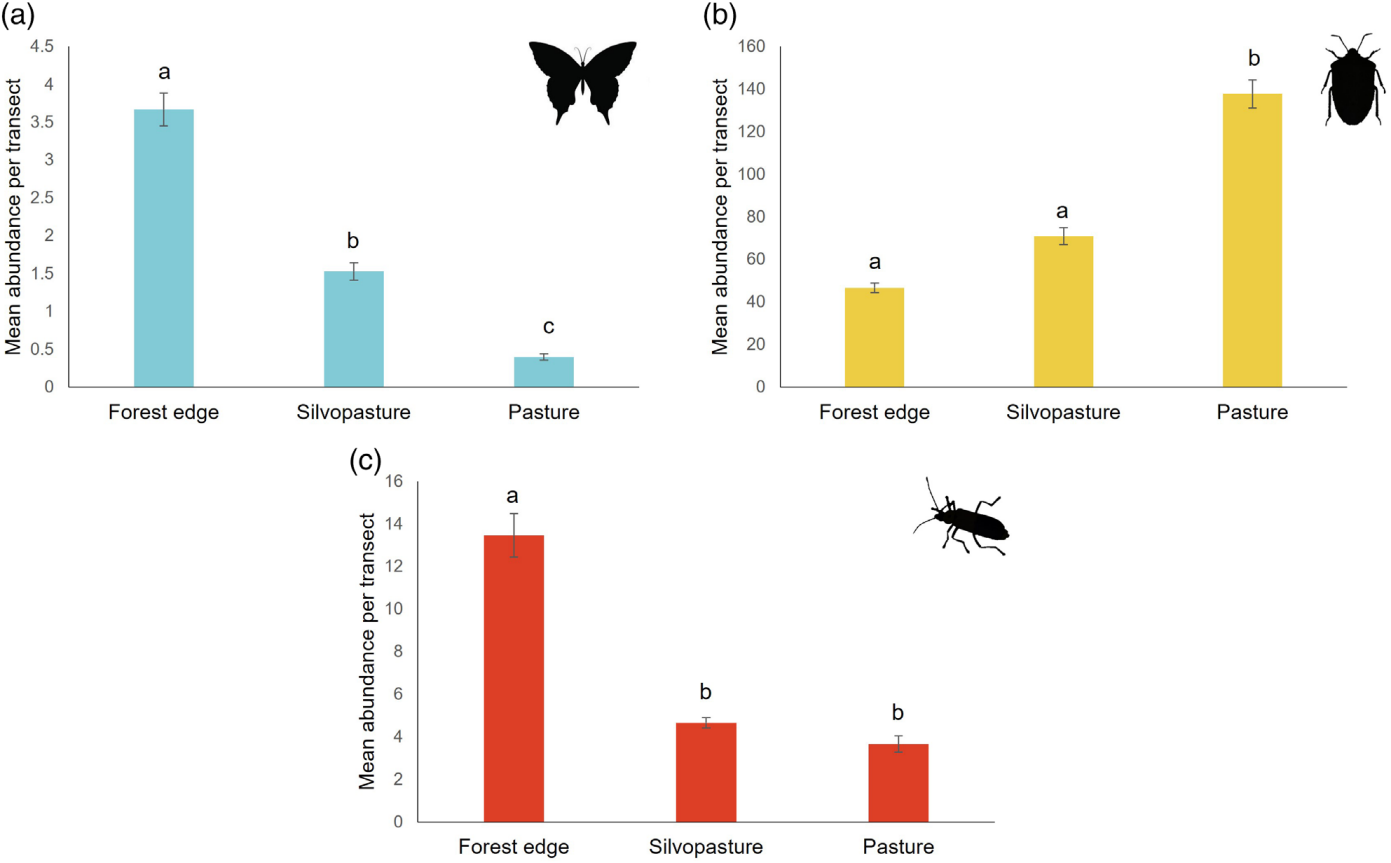
Bar plot showing mean abundance per transect ± standard error for (a) Lepidoptera, (b) Hemiptera and (c) Coleoptera according to habitat type. Univariate hypothesis testing detected significant differences in these orders according to habitat type (Table 1) different letters reflect significant differences (*p* ≤ 0.05) found during post hoc analysis comparing mean abundance across the different habitat types.

Following univariate hypothesis testing to compare community assemblages among habitats, there were clear contrasts in the abundance of different orders across each of the habitats. For sweep net collections, these differences were significant for Lepidoptera, Hemiptera and Coleoptera (Figure 4, Table 1). For the Lepidoptera, abundance varied significantly between all three habitat types. Greater numbers of Lepidoptera and Coleoptera were detected in forest edge habitats compared to traditional pasture. While greater Hemiptera abundance was observed in traditional pastures compared to forest edges and silvopasture (Table S6). For malaise trap collections, although differences were observed, these were not significant (Table 1).

**TABLE 1:** Results of univariate hypothesis testing for different invertebrate orders collected through sweep netting and malaise trapping from forest, traditional pasture and silvopasture habitats on smallholder livestock farms in Caquetá, Colombia.

	Sweep net		Malaise	
Order	Test	*p* value	Test	*p* value
Acari	0.024	0.981	NA	NA
Araneae	0.919	0.942	3.381	0.896
Blattodea	NA	NA	0.316	0.99
Coleoptera	**16.846**	**0.004**	5.221	0.741
Collembola	3.512	0.855	1.018	0.99
Diptera	0.384	0.958	0.245	0.99
Hemiptera	**15.363**	**0.012**	2.116	0.981
Hymenoptera	8.815	0.187	0.755	0.99
Lepidoptera	**19.251**	**0.001**	3.969	0.878
Odonata	2.157	0.916	NA	NA
Orthoptera	1.493	0.937	0.509	0.99
Phasmatodea	NA	NA	1.622	0.99
Psocoptera	2.343	0.916	1.558	0.99
Thysanoptera	3.894	0.826	NA	NA
Trichoptera	3.016	0.87	1.387	0.99

*Note*: The *p*‐values in bold indicate significant effects of habitat type on order abundance (*p* < 0.05).

## DISCUSSION

When comparing invertebrates collected on farms, we found that habitat type significantly affected invertebrate communities collected by sweep nets with the greatest contrast between forest edges and pastures. It appears that silvopasture could represent an intermediate habitat that supports invertebrate communities with similar components to both forest edges and traditional pasture. Some orders were more affected by habitat type in terms of abundance than others. While Lepidoptera and Coleoptera were most abundant at forest edges, Hemiptera were the most abundant group in traditional pastures. These findings have implications for the role of silvopasture for species conservation and ecosystem service delivery on livestock farms.

The invertebrate communities sampled at forest edges and in traditional pastures were most different in terms of community composition, diversity and evenness and abundance of particular orders and this is likely driven by two factors. Firstly, although still part of the pasture area, the forest edge survey transects included additional vegetation structure provided by shrubs and small trees that had been established at the forest edge. This more heterogeneous forest edge vegetation structure not only provided increased habitat complexity but also offered additional host plants for specialist species, which alters invertebrate communities and increases species richness (Borges & Brown, 2001; Reid & Hochuli, 2007). Secondly, by definition, the forest edge surveys were carried out in close proximity to areas of remnant forest found on each farm. Therefore, the invertebrate assemblage is likely affected by species that spill over from the forest habitats (Magura & Lövei, 2019), although this depends on their mobility (Gray et al., 2016). Interestingly, invertebrate communities found in silvopasture transects overlapped considerably with forest and traditional pasture and were not significantly different from either. This suggests that silvopasture is providing an intermediate habitat that can support invertebrate groups common to both pasture and forest. The introduction of trees into the pasture systems could increase structural complexity and provides additional hosts for enhancing invertebrate biodiversity (Jose et al., 2019; McAdam & McEvoy, 2009).

Creating multifunctional landscapes that deliver for production and conservation is a perennial challenge for global agricultural systems (Garibaldi et al., 2019; Kremen, 2020). Increasing habitat and structural complexity within the landscape matrix, as well as greater amounts of edge habitat and boundary features not only provides greater niche breadth for biodiversity (Garratt et al., 2017; Kremen, 2020) but also promotes delivery of ecosystem services in agroecosystems (Martin et al., 2019). Our study showed that Hemiptera were in greatest abundance in the traditional pasture sites. Hemipterans are predominantly herbivorous and comprise many pests of global importance. Aphids, hoppers and particularly spittlebugs are a key forage pests in tropical systems (Thompson, 2004; Valério et al., 2001). Traditional pastures likely provide a more suitable habitat for herbivorous sucking insects, compared to at the forest edges and in silvopasture areas where trees and shrubs provide structural complexity, both in terms of habitat and within‐plant architecture, factors which are known to increase natural invertebrate pest control (Langellotto & Denno, 2004). This may help reduce invertebrate herbivore abundance in silvopasture and forest edge habitats. Coleoptera, an incredibly diverse group, which includes some highly mobile predatory species, were also in greatest abundance in the forest edge habitats in our study. Coleoptera are known to spill over into pasture from woodland and may be helping reduce herbivore abundance (Magura & Lövei, 2019). However, newly established plantations of trees can negatively affect coleopteran species richness and abundance compared to natural forests and may explain why there was little difference between silvopasture and traditional pasture for this group in our study (López‐Bedoya et al., 2021).

Our study supports the necessity for using different invertebrate sampling methods to capture the full spectrum of the invertebrate community when seeking to compare habitats as invertebrates collected by sweep netting and malaise trapping were contrasting (Prado et al., 2017; Shweta & Rajmohana, 2016). That no significant differences between habitats for communities or orders were observed for Malaise traps catches could be because these traps collect more mobile or flying invertebrates, which respond to habitat cover and configuration at larger spatial scales (Martin et al., 2019). Nonetheless, the NDMS plots for malaise traps showed some similarities with those for sweep netting. While for some groups (e.g., Lepidoptera), the results from sweep netting and malaise trapping showed contrasting effects of habitat, these differences may be due to collecting different invertebrate species and life stages within the order Lepidoptera. The results for Hemiptera and Coleoptera collections in malaise traps and through sweep netting were similar.

Here we have explored invertebrate communities in different farmland habitats to understand how silvopasture adoption may shape invertebrate biodiversity and influence the ecosystem services they provide. However, without detailed taxonomic data to family or species level, it is not possible to fully understand the effects on biodiversity or the functional effects of differences in abundance among orders, which have considerable intrinsic diversity in species form and function. In our study, effects of silvopasture adoption on invertebrates were seen even though the trees were established only in 2016 and were not fully grown, and the silvopasture areas were relatively small when compared to other habitat types on the farms. We might expect to see effects increase as the trees grow and mature, or if the silvopasture plots had been larger and less susceptible to spill over from neighbouring habitats (Ries et al., 2004). Similarly, the trees themselves were not sampled directly for invertebrates as the canopy of trees is not effectively sampled by sweep netting. Contrasts between invertebrate communities between habitats with trees and those without would likely been even greater if this part of the habitat was also sampled.

We have been able to highlight differences between the invertebrate communities found in three different habitats on smallholder Colombian farms, and our results suggest silvopasture may act as an intermediate habitat for invertebrate assemblages between traditional pasture and forest. Thus, silvopasture has the potential for highly productive livestock farming (Jose & Dollinger, 2019), while also delivering benefits in terms of biodiversity and potential ecosystem services. When farmers were presented with our findings, they were particularly interested in the potential impact of some species on functions such as pest control or pasture protection, such findings could be used to promote wider adoption. Silvopastoral systems will never replace the unique biodiversity supported by native forests, so preventing deforestation and conserving undisturbed tropical forests should remain the priority. Silvopasture, however, could deliver part of the solution for arthropod conservation if integrated into landscape scale planning and management.

## AUTHOR CONTRIBUTIONS

**Lois Kinneen:** Data curation; formal analysis; investigation; methodology; writing – original draft; writing – review and editing. **Maria Paula Escobar Tello:** Conceptualization; funding acquisition; project administration; supervision; writing – review and editing. **Luis Miguel Hernandez:** Conceptualization; investigation; methodology; writing – review and editing. **Jill Thompson:** Conceptualization; funding acquisition; investigation; methodology; project administration; writing – review and editing. **Yardany Ramos‐Pastrana:** Data curation; investigation; supervision; writing – review and editing. **Eric Córdoba‐Suarez:** Data curation; investigation; writing – review and editing. **Miguel Romero‐Sanchez:** Conceptualization; investigation; methodology; project administration; supervision; writing – review and editing. **Andrew Barnes:** Conceptualization; funding acquisition; project administration; writing – review and editing. **Marcela Quintero:** Conceptualization; funding acquisition; project administration; supervision; writing – review and editing. **Michael Garratt:** Conceptualization; funding acquisition; investigation; methodology; project administration; supervision; writing – original draft; writing – review and editing.

## CONFLICT OF INTEREST STATEMENT

The authors declare no conflict of interest.

## Supporting information


**Data S1.** Supporting Information.

## Data Availability

Data are available from the University of Reading data archive. DOI: https://doi.org/10.17864/1947.000494.
