# Supplementary material for: Silvopastoral systems benefit invertebrate biodiversity on tropical livestock farms in Caquetá, Colombia
Source: Agric For Entomol. 2023 Oct 10;26(1):126–34. doi: 10.1111/afe.12594 (PMC10952216; doi:10.1111/afe.12594)
Supplement: Supplementary file 1 — Data S1. Supporting Information. [file AFE-26-126-s001.docx]

**Supplementary Material**

**Map of study site**


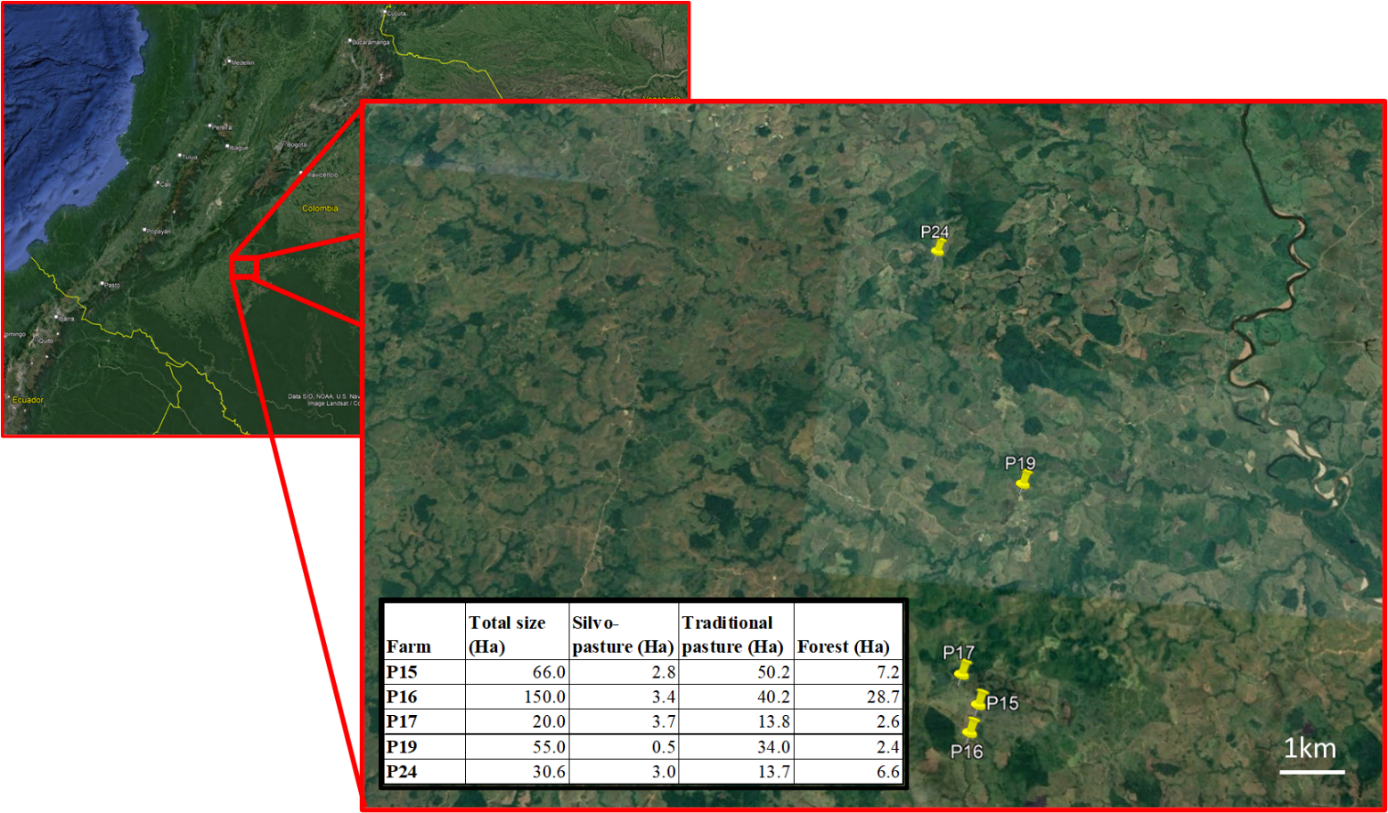


Figure S1 Location of the five study farms in the municipality of Morelia and Department of Caquetá in southern Colombia. Table includes the coverage in hectares per farm of each of the focal habitat types. Map created using Google Earth imagery accessed 2023.

**Ranked abundances of orders sampled by different methods**


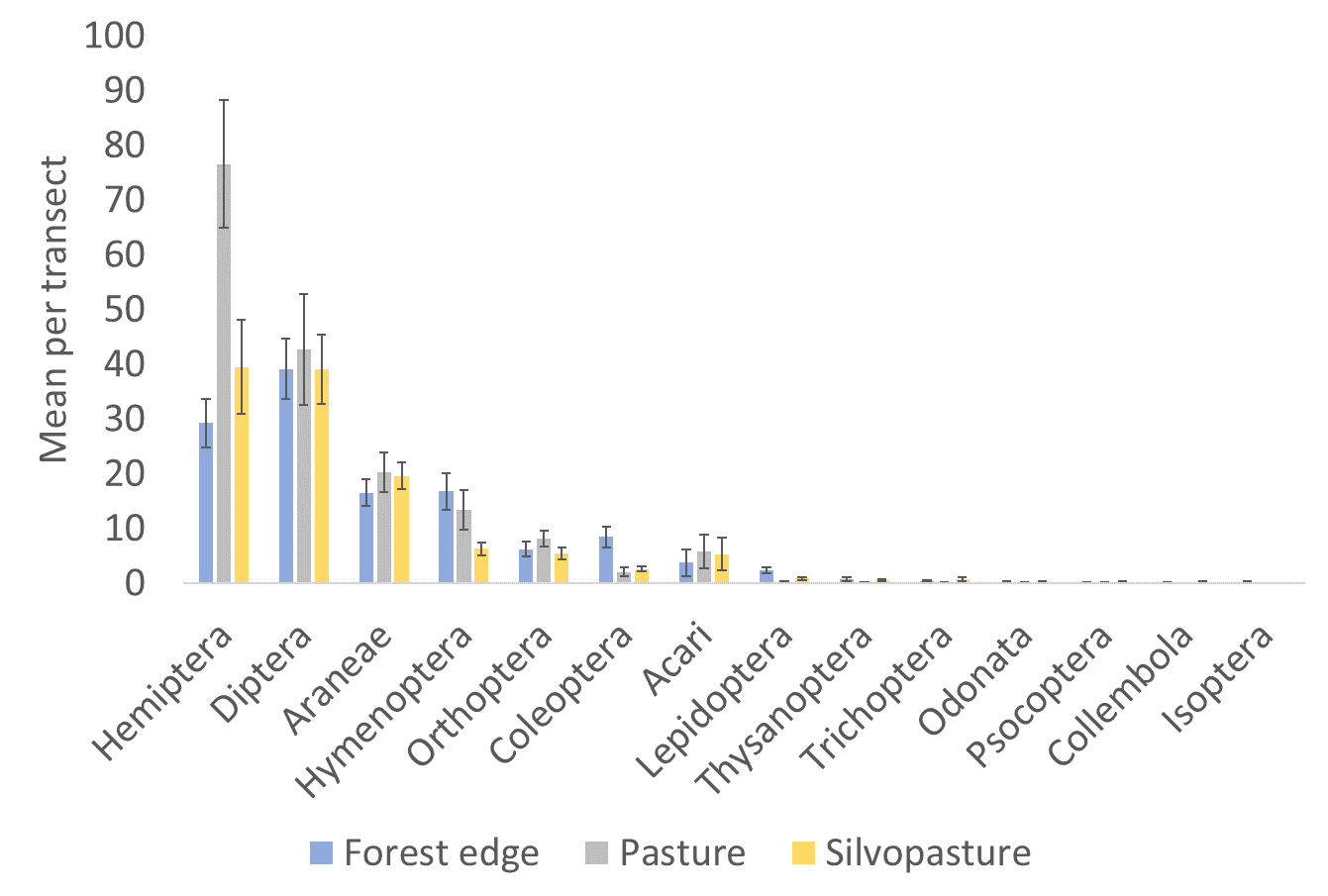


Figure S2 Ranked mean abundance (±SEM) by Order for invertebrates collected on sweep transects across five farms in Caquetá, Colombia. Isoptera was excluded from formal analysis as it was found only once across all transects.


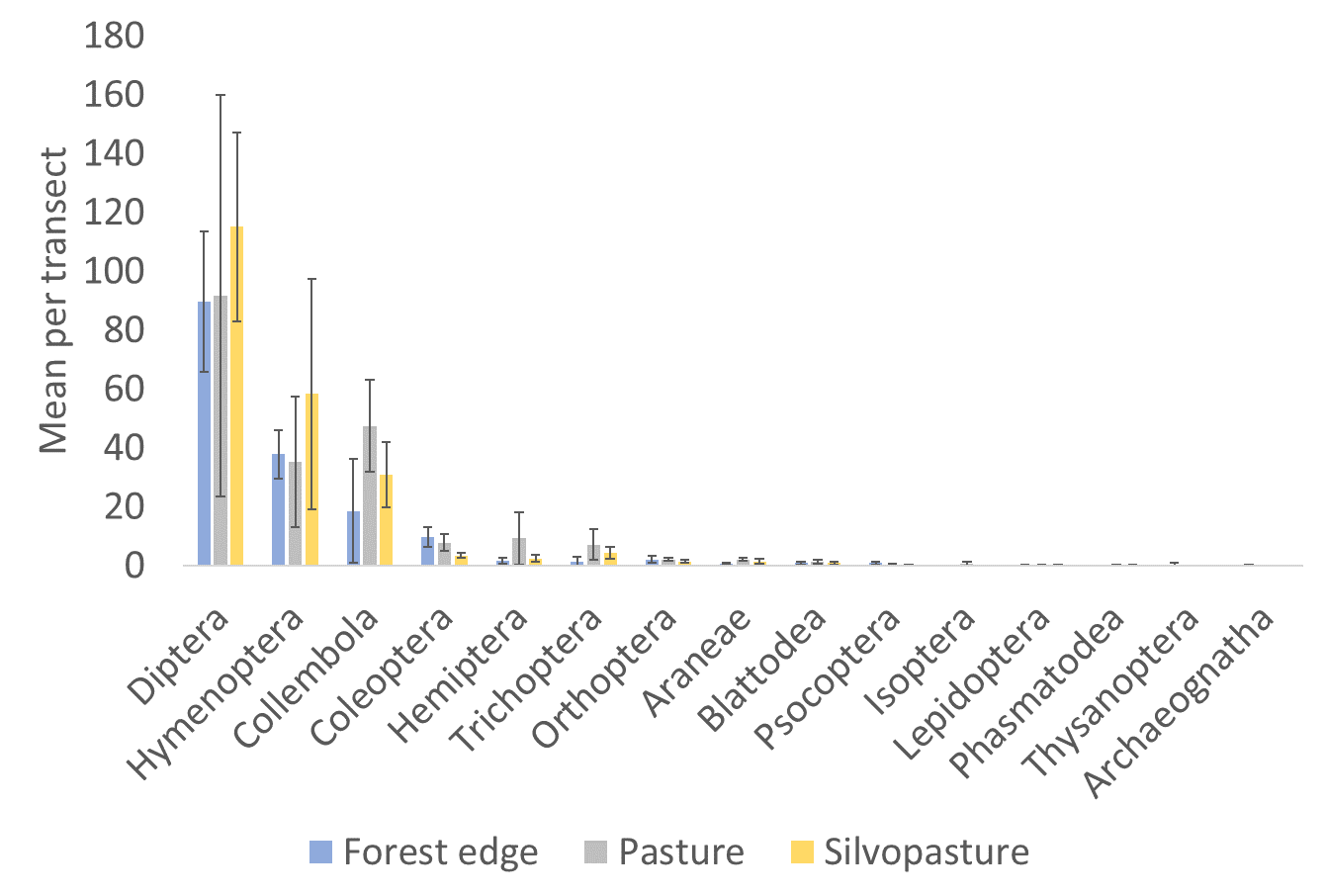


Figure S3 Ranked mean abundance (±SEM) by Order for invertebrates collected using malaise traps across five farms in Caquetá, Colombia. Archaeognatha, Isoptera and Thysanoptera were excluded from formal analysis as they were each collected only once during data collection.

*Table S1 Mixed model structure and results of analysis to compare the effects of habitat types on Order Richness, Shannon Diversity Index and Pielou’s evenness for invertebrate communities collecting using sweep netting.*

| **Order Richness** | | | | |
| --- | --- | --- | --- | --- |
| **Model structure:**  SwRichness<-glmer(Richness ~ Habitat + (1 \| Farm:Round), data = Sweeps, family=poisson) | | | | |
|  | Estimate | SE | z value | Pr(>\|z\|) |
| HabitatSilvopasture | -0.04 | 0.11 | -0.37 | 0.71 |
| HabitatPasture | -0.19 | 0.11 | -1.73 | 0.08 |
|  | | | | |
| **Shannon Diversity Index** | | | | |
| **Model structure:**  SwShannon<-lmer(H ~ Habitat + (1 \| Farm:Round), data = Sweeps) | | | | |
|  | Estimate | SE | t value |  |
| HabitatSilvopasture | -0.07 | 0.1 | -0.67 |  |
| HabitatPasture | -0.26 | 0.1 | -2.67 |  |
|  |  |  |  |  |
| **Pairwise Tukey Comparison** |  |  |  |  |
|  | Estimate | SE | d.f. | P value |
| Forest - Silvopasture | 0.07 | 0.1 | 67.4 | 0.78 |
| **Forest - Pasture** | **0.26** | **0.1** | **67.4** | **0.03** |
| Silvopasture - Pasture | 0.19 | 0.1 | 67.5 | 0.11 |
|  | | | | |
| **Pielou's Evenness** | | | | |
| **Model structure:**  SwEven <- lmer(J ~ Habitat + (1 \| Farm:Round), data=Sweeps) | | | | |
|  | Estimate | SE | t value |  |
| HabitatSilvopasture | -0.02 | 0.03 | -0.74 |  |
| HabitatPasture | -0.07 | 0.03 | -2.54 |  |
|  |  |  |  |  |
| **Pairwise Tukey Comparison** |  |  |  |  |
|  | Estimate | SE | d.f. | P value |
| Forest - Silvopasture | 0.02 | 0.03 | 64.8 | 0.74 |
| **Forest - Pasture** | **0.07** | **0.03** | **64.8** | **0.04** |
| Silvopasture - Pasture | 0.05 | 0.02 | 63.6 | 0.16 |

*Table S2 Results of non-parametric Kruskall-Wallis tests run to test for differences between Order richness, Shannon diversity Index and Pielou’s evenness in communities of invertebrates sampled using malaise traps.*

| **Metric** | **Χ^2^** | **d.f.** | **P- value** |
| --- | --- | --- | --- |
| Order richness | 0.4 | 2 | 0.82 |
| Shannon diversity | 4.34 | 2 | 0.15 |
| Pielou's evenness | 2.66 | 2 | 0.26 |

**Community comparisons**

**
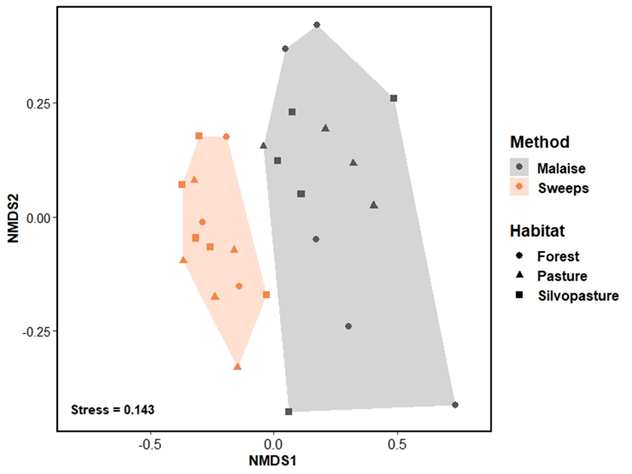
**

*Fig S4 NMDS plot representing invertebrate communities collected using sweep nets (orange) or malaise traps (grey) from three different habitat types located on smallholder livestock farms in Caquetá, Colombia. Points represent the community composition collected in different habitat types on five farms. The differences between communities were statistically significant (Wald value/test statistic 3.30, d.f. 28, P 0.004, Table S3).*

**Comparing differences in invertebrate communities according to sampling method: *mvabund* outputs**

Methods1<-manyglm(mvabundMethods ~ Method, family ="binomial")

summary(Methods1)

*Table S3 Model output using manyglm function to compare invertebrate communities collected using two sampling methods.*

| **Analysis of Deviance Table** | | | | |
| --- | --- | --- | --- | --- |
|  | Res.Df | Df.diff | Wald value | Pr(>wald) |
| (Intercept) | 29 |  | 4.66 | **0.01*** |
| Method | 28 | 1 | 3.30 | **0.004*** |
|  |  |  |  |  |

**Comparing invertebrate communities according to habitat type: *mvabund* outputs**

**Sweeps:**

Sw3<-manyglm(mvabundSweeps ~ Habitat, family ="negative_binomial", offset = Sweeps$No_Rounds, block=Farm)

Plot(Sw3)

summary(Sw3)

pairwiseSweeps<-anova(Sw3, pairwise.comp = Habitat, nBoot = 999, p.uni="adjusted")

pairwiseSweeps

*Table S4 Summary of model outputs comparing invertebrate communities sampled using sweep nets according to habitat, and post hoc pairwise comparison of differences between habitat types.*

| **Analysis of Deviance Table** | | | | |
| --- | --- | --- | --- | --- |
|  | Res.Df | Df.diff | Dev | Pr(>Dev) |
| (Intercept) | 44 |  |  |  |
| Habitat | 42 | 2 | 78.02 | **0.001*** |
|  |  |  |  |  |
| **Pairwise comparison results:** | | | | |
|  | Observed statistic | | Stepdown Adjusted P-value | |
| Forest vs. Pasture | 48.26 | | **0.013*** | |
| Pasture vs. Silvopasture | 33.63 | | 0.071 | |
| Forest vs. Silvopasture | 28.90 | | 0.072 | |

**Malaise:**

Mal2<-manyglm(mvabundMalaise ~ Habitat, family ="negative_binomial")

plot(Mal2)

summary(Mal2)

pairwiseMalaise<-anova(Mal2, pairwise.comp = Habitat, nBoot= 999, p.uni="adjusted")

pairwiseMalaise

*Table S5 Summary of model outputs comparing invertebrate communities sampled using malaise traps according to habitat and post hoc pairwise comparisons of differences between habitat types.*

| **Analysis of Deviance Table** | | | | |
| --- | --- | --- | --- | --- |
|  | Res.Df | Df.diff | Dev | Pr(>Dev) |
| (Intercept) | 14 |  |  |  |
| Habitat | 12 | 2 | 22.1 | 0.746 |
|  |  |  |  |  |
| **Pairwise comparison results:** | | | | |
|  | Observed statistic | | Stepdown Adjusted P-value | |
| Forest vs. Silvopasture | 14.19 | | 0.86 | |
| Pasture vs. Silvopasture | 9.65 | | 0.89 | |
| Forest vs. Pasture | 9.11 | | 0.89 | |

**Post-hoc pairwise comparisons between habitat types for Lepidoptera, Coleoptera and Hemiptera**

Significant effects of habitat type were detected for Coleoptera, Lepidoptera and Hemiptera collected using sweep nets during univariate hypothesis testing (See Table 1). These orders were then analysed separately and post-hoc analysis was carried out to understand the differences between habitat types.

*Table S6 Summary of model structures and post-hoc pairwise comparisons of abundance of Coleoptera, Lepidoptera and Hemiptera sampled using sweep nets between habitat types*

| **Coleoptera** | | |
| --- | --- | --- |
| Coleo1 <- glmer(Coleoptera~Habitat +(1 \| Farm:No_Rounds),data=Coleoptera, family=poisson) | | |
| **Pairwise habitat comparison results:** | | |
|  | Estimate | P value |
| Forest vs. Silvopasture | 1.07 | **<0.0001** |
| Pasture vs. Silvopasture | 1.31 | 0.37 |
| Forest vs. Pasture | 0.24 | **<0.0001** |
| **Lepidoptera** |  |  |
| Leps2 <- glmer.nb(Lepidoptera~Habitat +(1 \| Farm:No_Rounds),data=Leps) | | |
| **Pairwise habitat comparison results:** | | |
|  | Estimate | P value |
| Forest vs. Silvopasture | 0.88 | **0.04** |
| Pasture vs. Silvopasture | 1.34 | **<0.001** |
| Forest vs. Pasture | 2.21 | **0.03** |
| **Hemiptera** |  |  |
| Hemiptera1 <- glmer(Hemiptera~Habitat +(1 \| Farm:No_Rounds),data=Hemiptera, family=poisson) | | |
| **Pairwise habitat comparison results:** | | |
|  | Estimate | P value |
| Forest vs. Silvopasture | -0.33 | 0.39 |
| Pasture vs. Silvopasture | -0.64 | **0.02** |
| Forest vs. Pasture | -0.97 | **0.0004** |
